# Interspecific variation in mortality and growth and changes in their relationship with size class in an old‐growth temperate forest

**DOI:** 10.1002/ece3.7720

**Published:** 2021-06-12

**Authors:** Takashi Masaki, Ryo Kitagawa, Tohru Nakashizuka, Mitsue Shibata, Hiroshi Tanaka

**Affiliations:** ^1^ Forestry and Forest Products Research Institute Tsukuba Japan; ^2^ Kansai Research Center Forestry and Forest Products Research Institute Kyoto Japan; ^3^ Japan Forest Technology Association Tokyo Japan

**Keywords:** demographic synergy, demographic trade‐off, hierarchical modeling, Ogawa Forest Reserve, phylogenetically independent contrasts, transition probability

## Abstract

1. Understanding trade‐offs between demographic parameters is crucial when investigating community assembly rules in high‐diversity forests. To this end, we estimated mortality and growth parameters, and correlations among them, across entire size classes for 17 tree species (*Betula*, *Carpinus*, *Fagus*, *Quercus*, *Castanea*, *Acer*, *Cerasus*, *Swida*, *Kalopanax*, and *Styrax*) using a dataset over 18 years obtained from an old‐growth forest in Japan.
2. Size classes were represented by 12 categories determined by age, height, and diameter at breast height (DBH) from new seedlings to stems of DBH >85 cm. We derived the annual mortality and growth for each species and class using estimates of transition probabilities between classes. Trade‐offs or synergies in growth and survival among species per size class were analyzed with and without the inclusion of phylogenetic relationships.
3. Annual mortality showed U‐shaped patterns across size classes for species that could potentially reach a DBH ≥55 cm: 0.2–0.98 for seedlings, 0.002–0.01 at DBH 35–45 cm, and ca. 0.01 at DBH ≥55 cm. Other species demonstrated monotonically decreasing mortality toward specific maximum size classes. When phylogenetic information was included in analyses, the correlations between survival and growth changed across size classes were significant for some classes: As an overall tendency, synergy was observed in growth and survival for seedling to sapling classes, trade‐offs for juvenile to DBH 15–25 cm classes, and synergy again for larger classes. When phylogenetic information was not included, a significant trade‐off was observed only at DBH 5–15 cm.
4. *Synthesis*. Trade‐offs at intermediate classes imply differentiation in demographic characteristics related to life history strategies. However, evolutionarily obtained demographic characteristics are not substantial drivers of niche differentiation in the study area. The polylemma of mortality, growth, and other parameters such as the onset of reproduction may also be important factors driving species‐specific demographic traits.

## Graphical abstract

Annual mortality by each size class for major species in a temperate forest. They show U‐shaped patterns across life stages for species with maximum DBH >40 cm or monotonically decreasing patterns toward specific maximum size classes around DBH 30 cm.

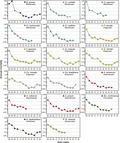

## 1 INTRODUCTION

Forest tree communities constitute assemblies of sympatric populations of long‐lived species. Understanding the drivers of community‐level processes requires close comparison of the demographic parameters among species (Benito‐Garzón et al., 2013; Kohyama et al., 2015). Simulation of long‐term changes in forests also requires knowledge of demographic parameters, particularly mortality (Bugmann et al., 2019). Tree mortality is important in that it can affect other trophic levels within forest communities. For example, the death of a large tree can lead to changes in the composition of soil microorganisms (Mueller et al., 2019), while also creating a habitat for wildlife (Franklin et al., 1987).

Previous studies, particularly those conducted in tropical forests, have reported trade‐offs between growth and survival (Kohyama et al., 2015; Philipson et al., 2014; Poorter et al., 2008; Wright et al., 2010). Understanding trade‐offs may be crucial when investigating community assembly rules in high‐diversity forests. Size dependency of demographic parameters has also been reported; U‐shaped mortality patterns with increasing tree diameter are well documented (Holzwarth et al., 2013; Hülsmann et al., 2016; Iida, Poorter, et al., 2014; Rüger et al., 2011). Tree size is considered exert a greater influence than cell senescence on the decline of radial growth and metabolism observed in aging trees (Mencuccini et al., 2005) and is likely strongly related to the higher mortality rates observed in larger trees. Demographic variations of this nature may play a role in determining niche coexistence among tree species in forest communities. Temporal variation in demographic parameters is also an important issue when considering climate change. For example, a century‐long dataset obtained from Swiss forests was used to relate annual climate variations to long‐term changes in tree mortality (Etzold et al., 2019). Where such long‐term data are not available, it is vital to understand current demographic parameters for future forest management.

Studies of tree demography in the temperate forests of East Asia are scarce, despite these forests’ relatively high diversity compared with those in other temperate regions (Latham & Ricklefs, 1993). This information gap prevents a comprehensive understanding of the demographics of the world's temperate forest communities. In addition, studies from temperate forests, including those in East Asia, have failed to assess demographic parameters for seedlings and saplings or have lacked analyses of large trees (Iida, Kohyama, et al., 2014; Iida, Poorter, et al., 2014; Rüger et al., 2011). Seedlings and saplings are highly vulnerable to herbivory and infection by pathogenic fungi under forest canopies (Shibata et al., 2010), and it is likely that demographic variation is likely to be profound in these early size classes. Additionally, with few exceptions (Clark & Clark, 1996; Hülsmann et al., 2016), the upper range of tree diameters typically sampled in previous studies rarely exceeded 50 cm, but mortality in larger trees has substantial ecological relevance, as stated above. Thus, comparative studies of tree demography should analyze entire life histories, from seedlings to old large trees. Finally, phylogenetic information has typically been overlooked in comparisons of demographic parameters among species. This has prevented an evolutionary understanding of niche assembly in terms of demography (e.g., synergies or trade‐offs between growth and survival). This may be because the inclusion of phylogenetic information in studies of tropical forests is impeded by exceptionally high diversity. However, phylogeny must be assessed to understand the evolution of demographic traits among species.

Here, we estimated nonspatial size class‐specific demographic parameters for 17 tree species in an old‐growth temperate forest in Japan using a dataset collected in 1987–2005. Seedlings and large trees (diameter at breast height [DBH] > 80 cm) were included in the dataset. Because DBH cannot be estimated for seedlings and saplings <1.3 m in height, because survivorship of current‐year seedlings is extremely low relative to older seedlings and because the heights of larger trees cannot be measured precisely, we used size class categories defined by age, height, and DBH to analyze the data inclusively (denoted simply as “size class” in this study), instead of a continuous measure of DBH used in previous studies (Iida, Kohyama, et al., 2014; Iida, Poorter, et al., 2014). Mortality, survival, and growth values for each class were derived from estimates of the probability of transitioning from that class. These estimates can be used in future studies as baseline parameters in the absence of disturbance related to climate change in the study area. We also analyzed correlations between growth and survival parameters for each size class with and without phylogenetic information. The former was used to test the influence of phylogenetic relationships on demographic parameters, and the latter was used to determine the ecological implications of demographic variation among species in the context of community assemblies. We addressed three research objectives: understanding how growth and mortality vary among species across size classes, identifying the classes at which survival and growth show synergy or trade‐offs, and determining how phylogenic relationships affect associations between survival and growth among species.

## 2 METHODS

### 2.1 Study site

The study site was located in the Ogawa Forest Reserve, an old‐growth deciduous forest located in the southern part of the Abukuma Mountains, Ibaraki Prefecture, central Japan (36°56′, 140°35′, 610 m in elevation). The mean monthly temperature is 10.7°C, with a high of 22.6°C in August and a low of −0.9°C in February. Annual precipitation is approximately 1,910 mm; August and September are the wettest months, and December and January are the driest. Maximum snow depth often reaches 50 cm (Mizoguchi et al., 2002). The dominant soil types are Andic Haplumbrepts and others (e.g., Andic Dystrochrepts). The forest canopy is mostly closed; only 9%–15% of the canopy was gap at the time of this study (Tanaka & Nakashizuka, 1997).

The study site, a 6‐ha permanent plot (300 × 200 m), was established in 1987. Within the plot, 61 woody species with free‐standing stems and girth at breast height (GBH) ≥15 cm were identified; 49 were tall species that can reach canopy strata, and 12 were tree and shrub species that cannot. The dominant taxa included members of Fagaceae (e.g., *Quercus serrata*, *Fagus japonica*), Betulaceae (e.g., *Carpinus laxiflora*, *Betula grossa*), and *Acer* (*A*. *pictum*) (Table 1, also see https://doi.org/10.5061/dryad.mpg4f4r05). Detailed information on the composition of woody species within the plot is available elsewhere (Masaki et al., 1992, 2015; Nakashizuka et al., 1992). The nomenclature follows the APG system.

**TABLE 1:** Sample size per size class, species‐specific maximum size class, and basal area in 1987 within the plot

Species	New seedling	Aged seedling	Sapling	Juvenile	D10–80	Maximum size class	Basal area (m^2^/ha)
Betulaceae							
*Betura grossa*	2,847	175	3	—	227	D80	0.58
*Carpinus cordata*	1,001	85	296	304	3,088	D50	0.68
*Carpinus japonica*	470	7	3	—	362	D40	0.28
*Carpinus tschonoskii*	5,319	21	30	16	565	D50	0.61
*Carpinus laxiflora*	7,090	74	71	98	3,020	D60	1.36
Fagaceae							
*Fagus crenata*	357	260	2,213	569	693	D80	2.77
*Fagus japonica*	2,273	96	493	192	3,947	D80	6.66
*Quercus crispula* var. *crispula*	48	18	27	—	275	D80	1.20
*Quercus serrata*	1896	348	15	—	1833	D80	8.58
*Castanea crenata*	87	42	13	—	371	D80	1.34
Rosaceae							
*Cerasus leveilleana*	171	48	27	2	459	D70	0.78
Sapindaceae							
*Acer amoenum*	2047	385	992	333	2,896	D60	0.92
*Acer rufinerve*	558	441	454	32	471	D30	0.36
*Acer pictum*	6,237	763	548	56	985	D80	0.89
Cornaceae							
*Swida controversa*	2092	103	96	11	823	D60	1.16
Sapindaceae							
*Kalopanax septemlobus*	300	33	129	84	104	D80	0.69
Styracaceae							
*Styrax obassia*	359	113	5	39	3,191	D30	0.80
Other species	1975	564	3,540	877	5,408	D80	2.69

Note

Size classes were defined as: new seedling (age <1 year), aged seedlings (age ≥1 year and height <30 cm), sapling (height 30 cm to 2 m), juvenile (height ≥2 m and DBH <5 cm), and D10–80 (DBH ≥5 cm). Dashes indicate that no observations were collected for that species at that class. The values of basal area are cited from Masaki et al. (1992) excepted for that of *Kalopanax septemlobus*, which was calculated from the unpublished data collected in 1987.

### 2.2 Tree inventory

We defined 12 categories based on age, height, and DBH: new seedling (age <1), aged seedling (age ≥1 and height <30 cm), sapling (height 30 cm to 2 m), juvenile (height ≥2 m and DBH <5 cm), D10 (DBH 5–15 cm, D10 was denoted by the midpoint of this range; later classes were similarly defined), D20, D30, D40, D50, D60, D70, and D80 (including trees with DBH ≥85 cm). These categories were denoted as “size classes” for simplicity because they were mostly defined by height or diameter. Data acquisition intervals were shorter for early classes, 1 or 2 years for the seedling to juvenile classes, and 2 or 4 years for sapling to D80 (Table S1).

Different sampling methods were used depending on size class. For classes D10–80, stems ≥15 cm GBH were identified, tagged, and then measured on seven occasions (May of 1987, 1989, 1991, 1993, 1997, 2001, and 2005; three datasets with 2‐year intervals and three with 4‐year intervals) to the nearest mm (Table S1). Survival was assessed during each measurement period. On each of the five occasions between 1989 and 2001, newly recruited stems ≥15 cm GBH were also tagged and then measured as described above in all subsequent visits. DBH was calculated by dividing the GBH by π, and data from stems with DBH ≥5 cm were provided for the analyses.

The 6‐ha plot was demarcated into 600 cells using a 10 × 10‐m grid; 2 × 2‐m quadrats were then placed at the cell's corners (651 quadrats, 2,604 m^2^ in total area). Saplings and juveniles were identified and tagged in the quadrats, and their heights were measured to the nearest cm. Inventories of the quadrats were conducted in May of 1987, 1989, 1990, 1992, 1994, 1996, 2000, and 2004 (one dataset with 1‐year intervals, four with 2‐year intervals, and two with 4‐year intervals). Between 1989 and 2000, new sapling recruits >30 cm in height were tagged (Table S1).

A 1‐ha subplot (100 × 100 m) was established at the center of the 6‐ha plot in 1988 and was demarcated into 400 cells using a 5 × 5‐m grid. In 1989, this subplot was enlarged to 1.2 ha (100 × 120 m), which included 480 grid cells. Small quadrats of 1 × 1 m in size were set up in about half of the corners of cells using a checkered pattern and were used to assess seedlings: 221 small quadrats were used in 1988, and 263 small quadrats were used in all subsequent years. New seedlings were tagged and identified during April and June within some or all of the small quadrats: 221 in 1988, 263 in 1989–1992, and 143 in 1993–2001 (Table S1). Seedling survival was assessed following their emergence throughout the study period. Then seedlings that survived their first year were classified as aged seedlings. Aged seedlings were also identified and tagged in small quadrats where new seedlings had not been tagged and identified in previous years (Table S1). Height and survival were assessed in May of every year from 1990 to 1999.

The stem densities of major species (see below) for each size class were calculated and are presented at https://doi.org/10.5061/dryad.mpg4f4r05.

### 2.3 Statistical analyses

As stated above, the datasets had different time intervals for some size classes. In these datasets, none of the tall tree species showed a transition of more than one class between consecutive measurements irrespective of interval length (exceptions were shrub species that often transitioned from seedling to juvenile stages within 2‐ or 4‐year intervals); this made it easy to estimate annual probabilities of transition between neighboring classes by using two or three of these datasets with different intervals. Furthermore, we assumed that the inventory data collected in each year were independent of those of another year to avoid overparameterization in statistical models (the effects of individual and survey years were not included in our analyses). Probabilities that were specific to species and size classes were assumed to be constant throughout the study period, and data obtained in different years were treated as replicates.

Among the 49 tall tree species found in the plot, we included species in which the total summed sample size over the study period exceeded five trees for the largest and second‐largest size‐class categories. These criteria led to 17 species being included in analyses of mortality and growth parameters (Table 1). Table 1 indicates the number of observations among species and size classes.

The low number of observations for some species in some size classes prevents the adoption of beta and log‐normal distribution models; these are often used as in Rüger et al. (2018). Instead, annual species‐specific transition probabilities between size classes were derived by using the datasets with 1‐, 2‐, or 4‐year intervals. We constructed a model linking multiple binomial probabilities simultaneously over different time intervals. Furthermore, we used a hierarchical Bayesian framework to estimate these probabilities; this enabled us to estimate parameters of species in classes that were lacking data (e.g., five species lacking juvenile‐class data) using data obtained from other species.

**FIGURE 1:**
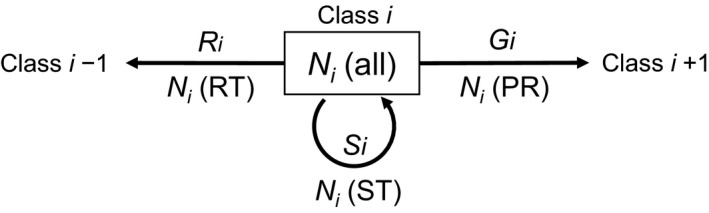
Notation of number of trees: *N*(ST) stagnating at a focal class (class *i* in this figure), and *N*(PR) and *N*(RT) respectively progressing and retrogressing from that class. The annual probability of stasis, progression, and retrogression are denoted by *S_i_*, *G_i_*, and *R_i_*, respectively. Annual mortality and growth were derived using 1 – *S_i_* – *G_i_* – *R_i_* and *G_i_*/(*S_i_* + *G_i_* + *R_i_*), respectively

The annual probabilities of a live individual stagnating in its current class *i* (i.e., stasis), advancing to the next class *i* + 1 by growth (i.e., progression), or moving back to the preceding class *i *− 1 (i.e., retrogression, assumed to be possible only for sapling and juvenile classes) were denoted as *S_i_*_,1_, *G_i_*_,1_, and *R_i_*_,1_, respectively (Figure 1), where subscript numbers represent the duration (i.e., 1 year). The value obtained from *G_i_*_,1_/(*S_i_*_,1_ + *G_i_*_,1_ + *R_i_*_,1_) (i.e., the probability of a living tree transitioning to the next size class) can be used as a substitute for the annual growth rate at class *i* (hereafter referred to as a “growth index” or simply “growth”). Annual survivorship was derived using *S_i_*_,1_ + *G_i_*_,1_ + *R_i_*_,1_, and annual mortality was obtained as 1—annual survivorship. The probabilities of stasis, progression, and retrogression can be decomposed into three probabilities: the probability of survival, *P*(SV), and two related conditional probabilities, *P*(ST or PR|SV) and *P*(ST|ST or PR), where SV, ST, and PR represent survival, stasis, and progression, respectively:

(1) $$\begin{array}{c} S_{i,1}=P_{i,1}\left(\text{SV}\right)\cdot P_{i,1}\left(\text{ST}\;\text{or}\;\text{PR}|\text{SV}\right)\cdot P_{i,1}\left(\text{ST}|\text{ST}\;\text{or}\;\text{PR}\right) \end{array}$$

(2) $$\begin{array}{c} G_{i,1}=P_{i,1}\left(\text{SV}\right)\cdot P_{i,1}\left(\text{ST}\;\text{or}\;\text{PR}|\text{SV}\right)\cdot \left(1‐P_{i,1}\left(\text{ST}|\text{ST}\;\text{or}\;\text{PR}\right)\right) \end{array}$$

(3) $$\begin{array}{c} R_{i,1}=P_{i,1}\left(\text{SV}\right)\cdot \left(1‐P_{i,1}\left(\text{ST}\;\text{or}\;\text{PR}|\text{SV}\right)\right) \end{array}$$

By transforming Equations (1)–(3), we obtain:

(4) $$\begin{array}{c} P_{i,1}\left(\text{SV}\right)=S_{i,1}+G_{i,1}+R_{i,1} \end{array}$$

(5) $$\begin{array}{c} P_{i,1}\left(\text{ST or PR}|\text{SV}\right)=\frac{S_{i,1}+G_{i,1}}{S_{i,1}+G_{i,1}+R_{i,1}} \end{array}$$

(6) $$\begin{array}{c} P_{i,1}\left(\text{ST}|\text{ST or PR}\right)=\frac{S_{i,1}}{S_{i,1}+G_{i,1}} \end{array}$$

The probability of survival and the two conditional probabilities in the above equations were estimated from the data shown in Figure 1 using the following statistical models based on binomial distributions:

(7) $$\begin{array}{c} N_{i,1}\left(\text{ST}\right)+N_{i,1}\left(\text{PR}\right)+N_{i,1}\left(RT\right)∼\text{Bin}\left(P_{i,1}\left(\text{SV}\right),N_{i,1}\left(\text{all}\right)\right) \end{array}$$

(8) $$\begin{array}{c} N_{i,1}\left(\text{ST}\right)+N_{i,1}\left(\text{PR}\right)∼\text{Bin}\left(P_{i,1}\left(\text{ST or PR}|\text{SV}\right),N_{i,1}\left(\text{ST}\right)+N_{i,1}\left(\text{PR}\right)+N_{i,1}\left(\text{RT}\right)\right) \end{array}$$

(9) $$\begin{array}{c} N_{i,1}\left(\text{ST}\right)∼\text{Bin}\left(P_{i,1}\left(\text{ST}|\text{ST or PR}\right),N_{i,1}\left(\text{ST}\right)+N_{i,1}\left(\text{PR}\right)\right) \end{array}$$

When 2‐year interval data were used, probabilities over 2 years at class *i* were linked to the 1‐year probabilities, as follows:

(10) $$\begin{array}{c} S_{i,2}=S_{i,1}^{2}+G_{i,1}\cdot R_{i+1,1}+R_{i,1}\cdot G_{i‐1,1} \end{array}$$

(11) $$\begin{array}{c} G_{i,2}=S_{i,1}\cdot G_{i,1}+G_{i,1}\cdot S_{i+1,1} \end{array}$$

(12) $$\begin{array}{c} R_{i,2}=R_{i,1}\cdot S_{i‐1,1}+S_{i,1}\cdot R_{i,1} \end{array}$$

Probabilities over 4 years at class *i* were linked to 1‐year probabilities via 2‐year probabilities:

(13) $$\begin{array}{c} S_{i,4}=S_{i,2}^{2}+G_{i,2}\cdot R_{i+1,2}+R_{i,2}\cdot G_{i‐1,2} \end{array}$$

(14) $$\begin{array}{c} G_{i,4}=S_{i,2}\cdot G_{i,2}+G_{i,2}\cdot S_{i+1,2} \end{array}$$

(15) $$\begin{array}{c} R_{i,4}=R_{i,2}\cdot S_{i‐1,2}+S_{i,2}\cdot R_{i,2} \end{array}$$

On the right side of Equations (10) and (13), the third term was eliminated to simplify the models given how rare this transition type was in the 2‐ to 4‐ year intervals, which resulted in the followings:

(16) $$\begin{array}{c} S_{i,2}=S_{i,1}^{2}+G_{i,1}\cdot R_{i+1,1} \end{array}$$

(17) $$\begin{array}{c} S_{i,4}=S_{i,2}^{2}+G_{i,2}\cdot R_{i+1,2} \end{array}$$

As stated earlier, we ignored the possibility that an individual could transition from class *i* to classes *i* + 2 or *i*–2 during 2‐ or 4‐year intervals to avoid highly complex models. Rates of stasis, progression, and retrogression over 2 or 4 years were also expressed by conditional probabilities over the same intervals, as in Equations (4)–(9). Estimates of these conditional probabilities obtained using data from 2‐ and 4‐year intervals were linked to annual probabilities using Equations ((11), (12), (14), (15), (16), (17)), ((11), (12), (14), (15), (16), (17)), and ((11), (12), (14), (15), (16), (17))–((11), (12), (14), (15), (16), (17)). Thus, data obtained from 1‐, 2‐, and 4‐year intervals were collectively used to estimate annual rates of stasis, progression, and retrogression and to derive growth and mortality.

For estimation, we introduced a hierarchical Bayesian framework separately for each size class, using species as a random effect to compensate for variation in sample size among species (Table 1). For example, the relationship between the number of stems $N_{i,1,k}\left(\text{ST}\right)$ and the 1‐year conditional probability $P_{i,1,k}\left(\text{ST}|\text{ST or PR}\right)$ at class *i* for species *k* was formulated by introducing the hyperparameters $\mu_{i,1}\left(\text{ST}|\text{ST or PR}\right)$ and $\sigma_{i,1}^{2}\left(\text{ST}|\text{ST or PR}\right)$:

(18) $$\begin{array}{c} N_{i,1,k}\left(\text{ST}\right)∼\text{Bin}\left(P_{i,1,k}\left(\text{ST}|\text{ST or PR}\right),N_{i,1,k}\left(\text{ST}\right)+N_{i,1,k}\left(\text{PR}\right)\right) \end{array}$$

(19) $$\begin{array}{c} \text{logit}\left(P_{i,1,k}\left(\text{ST}|\text{ST or PR}\right)\right)∼N\left(\mu_{i,1}\left(\text{ST}|\text{ST or PR}\right),\sigma_{i,1}^{2}\left(\text{ST}|\text{ST or PR}\right)\right) \end{array}$$

The same hierarchizations were introduced for the probability of survival and another conditional probability. Vague prior distributions were used for *μ_i_*_,1_ and $1/\sigma_{i,1}^{2}$, respectively; we used a normal distribution with a mean of 0 and a variance of 1,000 and a gamma distribution with both the shape and rate parameters set to 100.

Posterior values were sampled using a Markov chain Monte Carlo (MCMC) procedure of 300,000 steps, including 200,000 burn‐in steps, thinned every 20 steps, resulting in 5,000 posterior samples per chain. Calculations were implemented using OpenBUGS (Lunn et al., 2009). Three chains were run for each size class. In total, we used the mean of 15,000 samples as a parameter estimate. After some trial runs, the initial values of $\text{logit}(P_{i,1,k})$, *μ_i_*_,1_ and $1/\sigma_{i,1}^{2}$ were set to −11, −1, and 1, respectively. MCMC convergence was assessed visually, with reference to Gelman and Rubin's (1992) convergence diagnostic ($\hat{R}$); generally, good convergence was observed. Among 363 estimated probabilities (excluding those over specific maximum classes obtained from 408 probabilities [24 elements (*S*_2,1_–*S*_12,1_, *G*_1,1_–*G*_11,1_, *R*_3,1_–*R*_4,1_) × 17 species]), 2 parameters had an $\hat{R}$ slightly greater than 1.10 (1.11 and 1.13), but appeared to have good convergence.

### 2.4 Phylogenetic information and correlations between mortality and growth

We constructed a phylogenetic tree for 17 species based on R20120829 (the Angiosperm Phylogeny Group III super tree) in Phylomatic version 3 (http://phylodiversity.net/phylomatic/). Branch lengths were estimated from evolutionary age based on fossil records (Wikström et al., 2001) using the BLADJ algorithm in PYLOCOM version 4.2. (Webb et al., 2008).

After estimating growth and mortality as described above, phylogenetic signals were tested in each size class using the phylogenetic tree. For this, we calculated Pagel's *λ*, an index commonly used to detect phylogenetic signals in species traits (Pagel, 1999). In this test, both growth and mortality were logit‐transformed; neither showed a distribution significantly different from the normal distribution in each size class after the transformation (*p* = .09 –0.91). Thus, Pearson's coefficients of correlations between growth and survival (i.e., 1−mortality) were calculated after logit transformation for each size class, to test for the presence of a trade‐off or synergy between them in an evolutionary context using the phylogenetic tree and phylogenetically independent contrasts (PICs) (Harvey & Pagel, 1991). These analyses were performed in R version 3.6.1 using the function “phylosig” of the package “geiger” (version 2.0.7) (Harmon et al., 2020) and the function “pic” of the package “ape” (version 5.3) (Paradis & Schliep, 2018). In addition, correlations that did not employ PICs were also calculated using the function “cor.test” in an ecological context. Species with a total sample size <5 were excluded from these analyses as their corresponding values would likely shrink around hyperparameters, and indices of growth for new seedlings and the specific largest size class were not assessed because they would inevitably be derived as 1 and 0, respectively.

## 3 RESULTS

### 3.1 Mortality and growth

**FIGURE 2:**
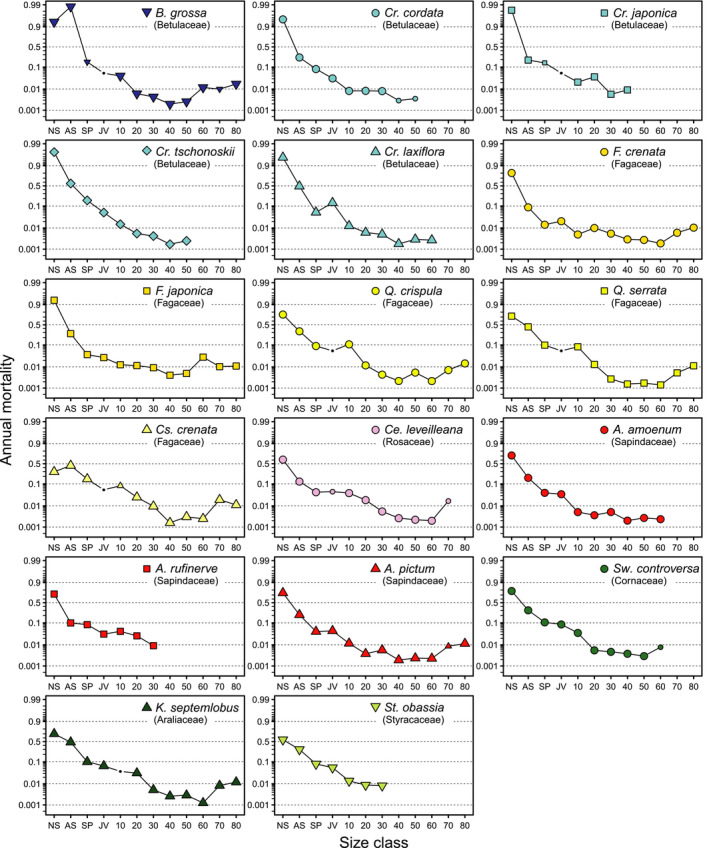
Annual mortality by each size class for 17 major species. Size classes were new seedling (age <1 year; NS), aged seedlings (age ≥1 year and height <30 cm; AS), sapling (height 30 cm to 2 m; SP), juvenile (height ≥2 m and DBH <5 cm; JV), D10 (5–15 cm in DBH), and later classes were defined similarly to D10 in 10‐cm intervals. The largest class, D80, included trees ≥85 cm DBH. Labels for D10–80 are shown using numeric characters without “D.” The y‐axes are scaled in logit. Smaller symbols represent species with a total sample size of 1–4 and dots represent no data wherein estimates corresponded with hyperparameters

Estimates of mortality for each size class are shown in Figure 2, and all estimates including those other than mortality are provided at https://doi.org/10.5061/dryad.mpg4f4r05. Broadly, annual mortality was greatest in the year following germination, ranging from 0.2–0.98. It then decreased monotonically to its lowest rate at D40–60 (approximately 0.002–0.01) and then increased to approximately 0.01 for species capable of reaching classes >D60. Species with smaller maximum sizes showed trends of monotonic decreases in mortality; mortality was lowest at D30–40, which corresponded with specific largest size class. In the new seedling to juvenile classes, species of Betulaceae showed relatively high mortality (a phylogenetic signal was significant), but differences between taxa became less distinct at later classes.

Unlike mortality, comparison of the growth index between classes was meaningless; the criteria for determining size‐class category differed among categories, as they were arbitrarily defined using age, height, and DBH. Thus, only within‐class comparisons among species were valid. These comparisons showed that species of Fagaceae generally had relatively higher growth in classes D10–40 and that species of *Carpinus* showed relatively lower growth in these classes (Figure S1) (a phylogenetic signal was significant at D40). This growth superiority of Fagaceae was lost at D50–80 (a phylogenetic signal was significant at D50, where Fagaceae growth tended to be lower). For classes prior to D10, no conspicuous patterns were observed among species (Figure S1).

### 3.2 Correlation between survival and growth

**FIGURE 3:**
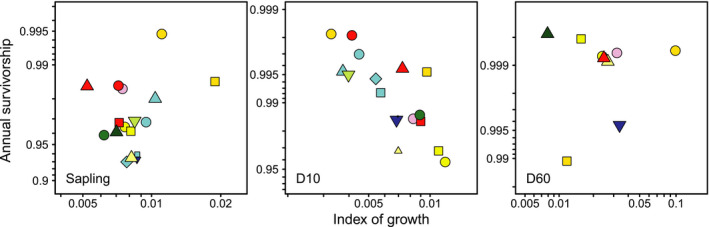
Relationships between annual survivorship and growth at the sapling, D10, and D60 classes, wherein statistically significant correlations were detected when phylogenetically independent contrasts were included. Colors and symbols follow Figure 1. Both the *x*‐ and *y*‐axes are scaled in logit

In this section, we use annual survivorship, instead of mortality to evaluate the relationship with growth. When phylogenetic information was included, correlations between survivorship and growth were substantial in some size classes (Table 2). In the aged seedling and sapling classes, survivorship and growth showed positive correlation coefficients, indicating synergy between them, but this was only marginally significant in the sapling class (Figure 3). In the juvenile–D20 classes, survivorship and growth showed negative correlation coefficients, indicating trade‐offs between them, but this relationship was only statistically significant in the juvenile and D10 classes (Figure 3). In later classes, they often showed positive correlation values; these were statistically significant at D60 and marginally significant at D30 (Figure 3). When phylogenetic information was not taken into consideration, correlations between survivorship and growth were only significant at D10, which was negative (*p* < .001), which indicates an ecological trade‐off between them. The other classes showed no correlations based on the absolute values of the coefficients >0.4 (*p* > .35).

**TABLE 2:** Pearson's coefficient of correlations between annual survivorship and growth obtained from analyses that included (left) and did not include (right) phylogenetic information

Life stage	Using PIC		Not using PIC		Number of species used for analysis
	Coefficient of correlation	*p* Value	Coefficient of correlation	*p* Value	
Aged seedling	0.346	.189	0.089	.733	17
Sapling	0.529	.052	0.325	.237	15
Juvenile	**−0.663**	.**037**	−0.098	.773	11
DBH 10	**−0.741**	.**002**	**−0.814**	.**000**	15
DBH 20	−0.358	.173	−0.238	.357	17
DBH 30	0.497	.071	0.022	.937	15
DBH 40	0.437	.155	0.006	.985	13
DBH 50	−0.022	.949	0.061	.852	12
DBH 60	**0.929**	.**001**	0.232	.549	9
DBH 70	0.056	.929	−0.243	.642	6

Note

Significant and marginally significant coefficients are shown in bold and underlined text, respectively. Species with a total sample size ≥5 per each class were used in these analyses, and the number of corresponding species are shown in the rightmost column.

## 4 DISCUSSION

This study revealed U‐shaped mortality–size relationships, implying congruence of the life history strategies of major species in the study area's tree community. Whereas, certain trade‐offs and synergies between survival and growth parameters were found in some classes when phylogenetic information was taken into account. This suggests the existence of variation in demographic characteristics among species. However, such evolutionarily obtained demographic characteristics did not appear to be substantial drivers of the niche differentiation in this temperate forest community. In the sections that follow, the study's findings are discussed in greater detail.

### 4.1 Variations in mortality among size classes

Among the species included in this study, U‐shaped mortality–size relationships were found for those with maximum size classes greater than D60 (i.e., *Betula*, *Fagus*, *Quercus*, *Castanea*, *Cerasus*, *Acer pictum*, and *Kalopanax*). Broadly, greater small tree mortality, lower medium tree mortality, and moderately greater mortality among large trees have also been observed in other studies (e.g., Holzwarth et al., 2013; Hurst et al., 2011; Lines et al., 2010). The minimum mortality of medium trees observed here (i.e., at the bottom of the U‐shape (0.002–0.008)), corresponded well with values reported from other forests (0.001–0.01), irrespective of climate, region, or continent (Gonzalez‐Akre et al., 2016; Holzwarth et al., 2013; Hülsmann et al., 2016; Hurst et al., 2011, 2012; Iida, Kohyama, et al., 2014; Iida, Poorter, et al., 2014; King et al., 2006; Ma et al., 2016; Muller‐Landau et al., 2006; Rüger et al., 2011; Ruiz‐Benito et al., 2013). Greater mortality in the seedling classes for species of Betulaceae likely reflects this family's typically small seed size, which is associated with the low resources within the seed used for the seedling (Shibata et al., 2010). This trait may disadvantage Betulaceae seedlings under old‐growth closed‐canopy conditions (Tanaka & Nakashizuka, 1997). By contrast, the low mortality of *Fagus crenata* in the seedling to D10 classes likely reflects the high shade tolerance of this species when it is young, and could allow it becoming a dominant canopy tree at the study site in the projected future (Masaki et al., 1992).

Other species showed monotonically decreasing patterns of mortality, with DBH being terminated at classes D30–60. Mortality values at species‐specific maximum size classes were not conspicuously different from those of the species discussed above for those same classes. This implies that the maximum size of these other species is determined by factors other than elevated mortality at maximum size. Their maximum size may be regulated by stasis, a potential cause of which is the onset of sexual reproduction under a closed canopy (Matsui, 1995; Suzuki et al., 2019).

Previous studies have shown that size trends for mortality that follow a U‐shaped pattern vary according to life form: small tree species reach their minimum at smaller sizes and tall tree species at larger sizes (King et al., 2006; Rüger et al., 2011). However, such patterns were not detected in this study, partly because we assessed tree species that had reached >30 cm in DBH. Many earlier studies analyzed trees reaching no more than 5–30 cm in DBH (King et al., 2006; Manso et al., 2015; Rüger et al., 2011). If we had included smaller tree species in the study forest (e.g., *Meliosma myriantha*, maximum DBH = 23 cm, *Acer carpinifolium*, maximum DBH = 12 cm, *Clethra barbinervis*, maximum DBH = 15 cm, or *Chengiopanax sciadophylloides*, maximum DBH = 18 cm; see Masaki et al., 2017) in our analysis, different mortality–size relationships may have been observed.

A unique element of this study was that we estimated the mortality rate among large trees (i.e., >D60), which we found to be around 0.01 per year. Owing to the small sample size and shrinkage effects unique to the hierarchical Bayesian framework, these estimates were similar among species. Thus, it was difficult to assess differences among species in terms of mortality at large sizes, but we believe that these general estimates provide valuable reference data. Greater mortality (i.e., 0.01–0.09 per year) has been reported in earlier research on trees >D60 in temperate forests (Gonzalez‐Akre et al., 2016; Hülsmann et al., 2016; Hurst et al., 2011). Compared with these estimates, large trees at our study site appeared to exhibit higher survivorship, potentially due to the closed canopy (Tanaka & Nakashizuka, 1997), which may support the survival of large trees (Hurst et al., 2011).

Although specific causes of mortality were not assessed in this study, the first 4 years of data obtained from the study site suggested that >70% of the mortality among small trees (<50 cm in DBH) was due to withering, and more than half of the observed mortality of large trees (≥50 cm DBH) resulted from catastrophic stem breakage (Nakashizuka et al., 1992). These patterns are concordant with those obtained in other forests: competition with or suppression by other trees is a major cause of mortality among smaller trees (Hurst et al., 2011; Sims et al., 2014), and wind events are a dominant cause of mortality among larger trees (Holzwarth et al., 2013). Damage by either insects or pathogenic fungi (Das et al., 2016; Sims et al., 2014) is also a major contributor to mortality among large trees and likely explains the mortality that we observed among trees >D60 in our study plot not attributed to catastrophic damage. Mortality resulting from drought stress (Stephenson et al., 2019) was unlikely in this study, because the study period did not include any year wherein annual precipitation was <1,000 mm (mean ± *SD* = 1,416 ± 238 mm, data obtained from Higashishirakawa Meteorological Station, located 16 km west of the study site; Japan Meteorological Agency, 2020). This amount of precipitation likely provided sufficient water for growth. Future research should further investigate the relationship between tree size and mortality causes.

### 4.2 Relationship between survival and growth

We observed significant growth–survival relationships in some size classes when phylogenetic information was incorporated into the analyses. Relationships showed positive values in the seedling–sapling classes (marginally significant for saplings) and in the D30–70 classes (significant at D60 and marginally significant at D30), which reflected synergy between growth and survival in these classes. By contrast, the relationship showed negative values in intermediate classes (significant for the juvenile and D10 classes), suggesting trade‐offs between growth and survival therein. Thus, as an overall tendency, the relationship between growth and survival changed from synergies to trade‐offs and back to synergies according to size class. To the best of our knowledge, such ontogeny‐related changes in growth–survival relationships are more complicated than reported elsewhere (McMahon et al., 2011; Rüger et al., 2018).

In the closed‐canopy study forest (Tanaka & Nakashizuka, 1997), small trees mostly occurred under the canopy, with few individuals present in canopy gaps. This may explain the observed positive correlation (synergy) between growth and survival in the vulnerable seedling and sapling classes (marginally significant in the latter class). The low‐light conditions under the closed canopy are not likely to be advantageous in terms of growth and survival for small individuals of shade‐intolerant species, such as *Betula*, *Quercus*, *Castanea*, and *Swida* (cf. Masaki et al., 1992). By contrast, shade‐tolerant species, such as *Fagus* and *A*. *pictum*, may not be disadvantaged under such conditions.

A trade‐off between growth and survival for trees has been reported in tropical and subtropical forests (Iida, Kohyama, et al., 2014; Iida, Poorter, et al., 2014; Kitajima & Poorter, 2008; Wright et al., 2003) and in temperate forests (Kunstler et al., 2009). In our study, statistically significant trade‐offs (negative correlations between growth and survival) were found from the juvenile to D20 size classes (significant in the juvenile and D10 classes). On average, trees in the study area with a DBH of 10 cm are approximately 10 m tall (Masaki et al., 2017). These trees form a subcanopy layer and are typically nearly half the height of the overstory canopy (Suzuki et al., 2019). This implies that tree species in the study area may differ in terms of their regeneration traits most markedly around the midpoint of their height growth course.

Our finding of a positive correlation between growth and survival for trees >D30 (marginally significant at D30 and significant at D60) is noteworthy and may reflect the maximum size per species. Within the family Betulaceae, the maximum size of *Carpinus cordata* and *Carpinus japonica* at D30–50 was smaller than that observed in other *Carpinus* species and *Betula grossa* (i.e., D50–80); we observed lower survivorship and growth rates in the former two species within the size classes just preceding their specific maximum classes. Similar patterns were observed within the genus *Fagus*. Overall synergy of growth and survival at sizes >D30 may therefore be constrained by phylogenetics. This synergy may come at the expense of reproduction (Shibata & Tanaka, 2002; Suzuki et al., 2019). The synergies and trade‐offs found in this study should be tested to assess the polylemma of growth, survival, reproduction, etc. (Rüger et al., 2018).

Synergies and trade‐offs were not significant when phylogenetic information was not included in the analyses, except for a trade‐off observed within D10. This indicates that evolutionarily obtained synergies and trade‐offs do not function effectively in niche differentiation, at least in terms of regeneration. In the stable old‐growth forest assessed here, a differentiated species‐specific life history trait was only apparent in the D10 class in an ecological context. Mechanisms promoting coexistence among species in the evolutionary context of demographic trade‐offs may play limited roles at our study site, which is species‐rich; other mechanisms, such as neutral processes or stochasticity in dispersal, may play more substantial roles (Masaki et al., 2015, 2019).

### 4.3 Future directions

We identified several avenues for future research on tree population dynamics in temperate forests. In the short term, one interesting issue is how species‐specific variations in demographic parameters may be tied to functional traits (Greenwood et al., 2017). Specifically, wood density is often closely correlated with mortality (Philipson et al., 2014; Poorter et al., 2008; Wright et al., 2010) and growth (Iida, Poorter, et al., 2014; Poorter et al., 2008). Studies that have reported associations between functional traits and demographic parameters have mostly been conducted in tropical forests, with limited examples from temperate forests. This information gap could be filled by studies utilizing functional trait data (Kunstler et al., 2016). In addition, within‐population variation should also be assessed in future work. Tree mortality can vary within populations of conspecifics depending on environmental conditions; trees may grow slowly due to competition, lower light availability, lower site productivity, and other factors, and mortality would likely be higher under these conditions (Vanoni et al., 2019). To provide general background parameters, our study focused on interspecific variations in demographic parameters, but we did not assess intraspecific differences. Future studies providing more precise parameter estimates should consider variations among subpopulations to assess population dynamics, which are likely influenced by spatial heterogeneity in light and site conditions (Abe et al., 2013; Kaneko & Kawano, 2002; Russo et al., 2007; Tanaka et al., 2008).

Our study assessed demographic data collected over an 18‐year period from an old‐growth temperate forest. The data were analyzed to generate background parameters that can be used, in the long term, to detect the effects of climate change on future forest dynamics. Several studies have reported an increase in tree mortality over the last 40–50 years, likely owing to drought stress (van Mantgem et al., 2009; McDowell et al., 2018; Peng et al., 2011; Smith et al., 2015). Unfortunately, such long‐term data are not available in East Asia, with the exception of some inventory data collected from commercial plantations (Matsushita et al., 2015). In East Asia, typhoon frequency is expected to increase in response to climate change (IPCC, 2014). This will likely lead to greater mortality among large trees, and consequently, various aspects of forest ecosystems may change in this region. Data on forest dynamics, such as those used in this study, are important for evaluating ecosystem‐level changes. Long‐term changes in forest systems are often the product of internal processes within forest communities such as increases in the number of large trees (Luo & Chen, 2015) or intensified competition between trees over time (Bradford & Bell, 2017). Furthermore, the effects of changes in temperature and precipitation on tree demography are likely to vary depending on species and site conditions (Etzold et al., 2019; Ruiz‐Benito et al., 2013). Therefore, tree population dynamics should be assessed by disentangling the effects of multiple external factors, such as variations in climate and site conditions, and internal factors, such as species identity and spatial patterns, to gain a more precise understanding of forest systems in East Asia.

## CONFLICT OF INTEREST

The authors declare no conflicts of interests.

## AUTHOR CONTRIBUTION

**Takashi Masaki:** Conceptualization (lead); Data curation (supporting); Formal analysis (lead); Funding acquisition (lead); Investigation (equal); Methodology (equal); Project administration (lead); Writing‐original draft (lead). **Ryo Kitagawa:** Formal analysis (equal); Writing‐original draft (supporting). **Tohru Nakashizuka:** Investigation (equal); Methodology (lead); Supervision (lead); Writing‐original draft (supporting). **Mitsue Shibata:** Data curation (lead); Investigation (equal); Writing‐original draft (supporting). **Hiroshi Tanaka:** Investigation (equal); Methodology (equal); Writing‐original draft (supporting).

## Supporting information

Supplementary Material

## Data Availability

https://doi.org/10.5061/dryad.mpg4f4r05.
