# Supplementary material for: Interspecific variation in mortality and growth and changes in their relationship with size class in an old‐growth temperate forest
Source: Ecol Evol. 2021 Jun 12;11(13):8869–81. doi: 10.1002/ece3.7720 (PMC8258222; doi:10.1002/ece3.7720)
Supplement: Supplementary file 1 — Supplementary Material [file ECE3-11-8869-s001.docx]

Fig. S1. Annual growth rates standardized for each size class for 17 major species. Size classes were new seedling (age < 1 year; NS), aged seedlings (age ≥ 1 year and height < 30 cm; AS), sapling (height 30 cm to 2 m; SP), juvenile (height ≥ 2 m and DBH < 5 cm; JV), D10 (5–15 cm in DBH), and later classes were defined similarly to D10 in 10-cm intervals. The largest class, D80, included trees ≥ 85 cm DBH. Labels for D10–80 are shown using numeric characters without “D.” Smaller symbols represent species with a total sample size of 1–4 and dots represent no data wherein estimates corresponded with hyperparameters.


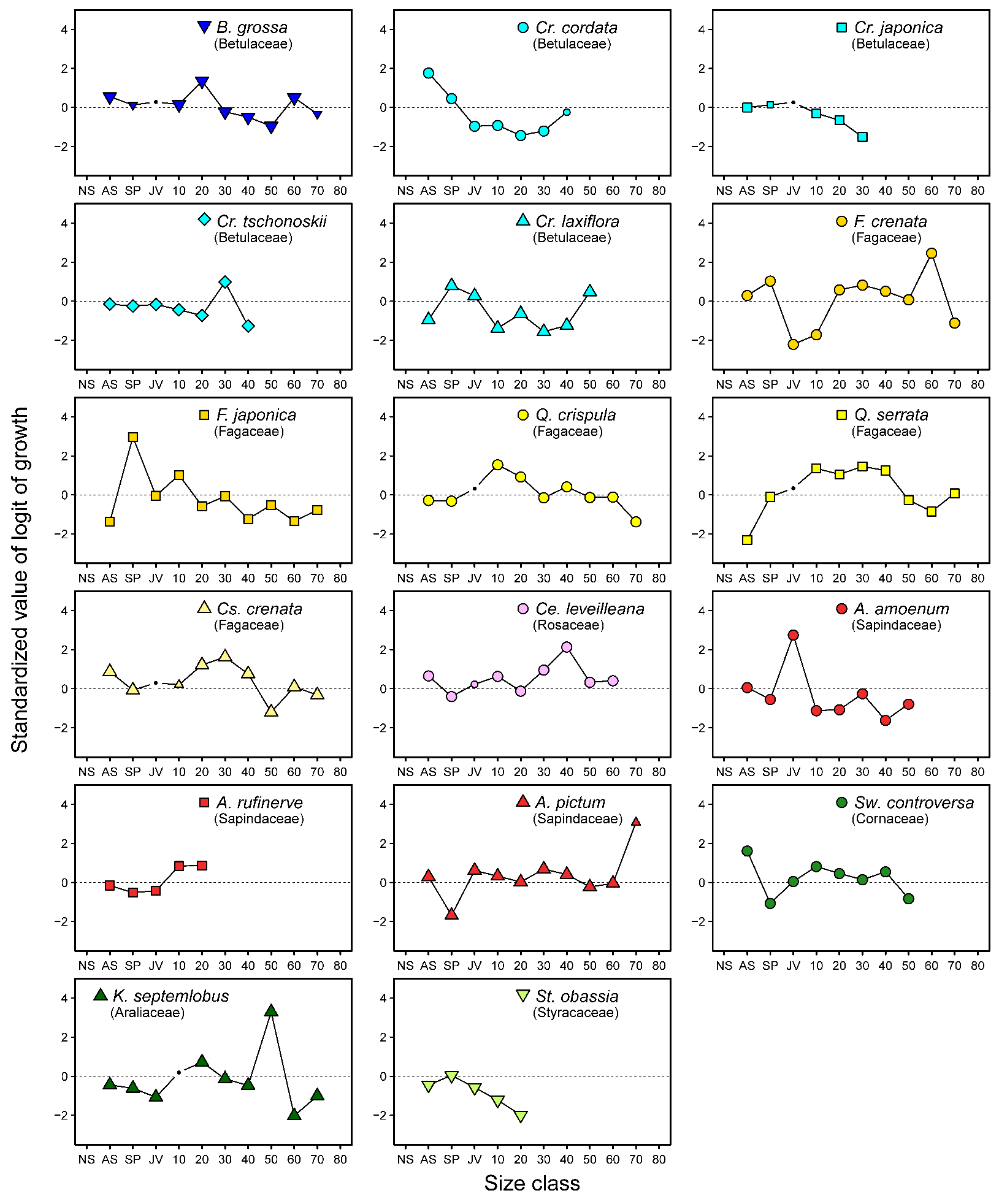


Table S1. The number of trees sampled within each size class in each year during the study period. Numbers in parentheses represent the interval, in years, until the next assessment. Size class were defined as follows: new seedling (age < 1 year), aged seedlings (age ≥ 1 year and height < 30 cm), sapling (height 30 cm to 2 m), juvenile (height ≥ 2 m and DBH < 5 cm), and D10–80 (DBH ≥ 5 cm). The smaller sample sizes for aged seedlings in 1989, saplings in 1987 and 1989, and juveniles in 1989 were due to the smaller number of quadrats assessed in these years.

| Year | New seedling | | Aged seedling | | Sapling | | Juvenile | | D10–80 | |
| --- | --- | --- | --- | --- | --- | --- | --- | --- | --- | --- |
| 1987 |  |  |  |  | 467 | (2) | 437 | (2) | 4790 | (2) |
| 1988 | 1789 | (1) |  |  |  |  |  |  |  |  |
| 1989 | 5491 | (1) | 53 | (1) | 632 | (1) | 212 | (1) | 4794 | (2) |
| 1990 | 1372 | (1) | 385 | (1) | 1522 | (2) | 396 | (2) |  |  |
| 1991 | 5460 | (1) | 287 | (1) |  |  |  |  | 4811 | (2) |
| 1992 | 1399 | (1) | 284 | (1) | 1520 | (2) | 397 | (2) |  |  |
| 1993 | 98 | (1) | 480 | (1) |  |  |  |  | 4835 | (4) |
| 1994 | 4703 | (1) | 347 | (1) | 1583 | (2) | 377 | (2) |  |  |
| 1995 | 4245 | (1) | 403 | (1) |  |  |  |  |  |  |
| 1996 | 3287 | (1) | 430 | (1) | 1739 | (4) | 389 | (4) |  |  |
| 1997 | 904 | (1) | 506 | (1) |  |  |  |  | 4741 | (4) |
| 1998 | 1129 | (1) | 401 | (1) |  |  |  |  |  |  |
| 1999 | 385 | (1) |  |  |  |  |  |  |  |  |
| 2000 | 156 | (1) |  |  | 1492 | (4) | 405 | (4) |  |  |
| 2001 | 4709 | (1) |  |  |  |  |  |  | 4747 | (4) |
